# Impact of the Anti‐Homosexuality Act on HIV service delivery in Uganda: Evidence from community‐led monitoring

**DOI:** 10.1002/jia2.70030

**Published:** 2025-09-12

**Authors:** Allan Nsubuga, Frank Mugisha, Beatrice Ajonye, Kenneth Mwehonge, Elise Lankiewicz, Patrick Drake, Esther Joan Kilande, Alice Kayongo, Alana R. Sharp

**Affiliations:** ^1^ Sexual Minorities Uganda (SMUG) Kampala Uganda; ^2^ International Community of Women Living with HIV ‐ Eastern Africa (ICWEA) Kampala Uganda; ^3^ Health Promotion and Social Development (HEPS) Kampala Uganda; ^4^ Andelson Office of Public Policy amfAR Washington DC USA; ^5^ Data, Et cetera Washington DC USA; ^6^ O'Neill Institute for National and Global Health Law Georgetown University Washington DC USA; ^7^ School of Public Health Georgetown University Washington DC USA

**Keywords:** Anti‐Homosexuality Act, community‐led monitoring, criminalization, men who have sex with men, policy impact, Uganda

## Abstract

**Introduction:**

In 2023, the Ugandan government enacted the Anti‐Homosexuality Act (AHA), which included expanded and intensified criminal penalties for consensual same‐sex relations. While arrests, harassment and violence have been reported, evidence of the AHA's impact on HIV healthcare delivery is limited. Community‐led monitoring (CLM) is an accountability mechanism that uses community‐gathered evidence to advocate for improved healthcare quality and is well‐positioned to describe changes in access and quality of care.

**Methods:**

Data from the CLM programme in Uganda were used to identify changes in healthcare delivery and use related to the AHA. As part of the CLM programme, routine survey data were collected from clients and managers in 320 public health facilities and 50 drop‐in centres (DICs) from 2022 to 2024. Survey data were analysed using a difference‐in‐differences logistic model to measure changes in indicator measures before and after the AHA was signed into law. Seven semi‐structured individual interviews were conducted with DIC facility managers, deductively coded and thematically analysed.

**Results:**

In public health facilities and DICs, the proportion of respondents identified as men who have sex with men (MSM) declined significantly after AHA. In facilities, all categories of key populations (KPs) reported high levels of discrimination. After the AHA, MSM reported significant reductions in key HIV‐related services compared to other populations, including lower rates of pre‐exposure prophylaxis (PrEP) counselling, lower participation in support groups and having fewer friendly staff interactions. In DICs, all types of clients were less likely to be referred to health facilities, receive PrEP and find the DIC easy to access after the AHA was signed. DIC managers described experiencing harassment, violence and staffing challenges due to AHA, which they responded to by leveraging partnerships with local and global allies, providing virtual services, and seeking registration as full‐service clinics.

**Conclusions:**

Data from the Uganda CLM programme provide an early view of the impact of the AHA on service delivery in public health facilities and DICs. While DICs and health facilities developed strategies to build resiliency and adapt, the AHA created significant barriers to care. These findings provide empirical warnings of the barriers experienced by KPs when accessing healthcare services in a criminalized context.

## 1 INTRODUCTION

Thirty countries in Africa criminalize consensual same‐sex sexual acts [1]. In Uganda, same‐sex acts have been criminalized for decades, with severe penalties up to life imprisonment [2]. In May 2023, President Yoweri Museveni signed the Anti‐Homosexuality Act (AHA), which expanded the scope of criminalization by introducing the death penalty for acts of “aggravated” homosexuality and broadly criminalizing activities “promoting” homosexuality [3]. While a similar act was passed in 2014 but annulled by the Constitutional Court of Uganda, the 2023 AHA was upheld in court, although several prohibitions were struck from the law, including the criminalization of HIV transmission, the duty to report and the prohibition of renting property to LGBTQ+ persons [4]. The AHA has drawn condemnation and sanctions from human rights and HIV advocates [5, 6, 7, 8], governments [9] and international institutions [10, 11].

Men who have sex with men (MSM) have a 23‐fold higher relative risk of HIV acquisition, as compared to the wider population [12]. While HIV incidence has declined globally by 39% since 2010, rates of new acquisitions among MSM have increased in the same period [12, 13]. Ending HIV as a public health threat is dependent on HIV services being accessible to MSM, yet laws that criminalize homosexuality contribute to stigma and discrimination [14, 15], create fear of and resistance to engaging with medical care [16], and are associated with worse HIV outcomes [17, 18].

Reports and leaked documents have revealed arrests, torture, forced evictions, targeted investigations of non‐governmental organizations (NGOs) and a chilling effect on advocacy related to the AHA [19, 20, 21, 22]. To mitigate the law's impact on healthcare, the Ministry of Health (MOH) issued a circular in June 2023 urging public health facilities to provide non‐discriminatory healthcare services to all clients [23].

With the AHA entering its second year, measuring the impact of the AHA on HIV service delivery is an urgent need. A consortium of community organizations has implemented a community‐led monitoring (CLM) programme since 2020, with Sexual Minorities Uganda (SMUG) leading the CLM efforts for key populations (KPs), in an effort funded by the U.S. President's Emergency Plan for AIDS Relief (PEPFAR) and supported by the Joint United Nations Programme on HIV/AIDS (UNAIDS). CLM is a social accountability tool in which trained healthcare service users collect data on the quality and availability of services in clinics and communities, which are then used to advocate with clinicians, governments and donors for improvements [24]. Routinely collected CLM data are used to describe the impact of AHA on service delivery.

## 2 METHODS

### 2.1 Data collection

This study analysed survey data and interviews collected by the CLM programme. On a quarterly basis, the programme gathered survey data from clients and managers at both public health facilities and drop‐in centres (DICs), which are facility‐ or community‐based spaces for KPs to receive commodities, health information, referrals, and, in some centres, clinical services. In addition to site‐based surveys, MSM, sex workers, transgender individuals and people who use drugs were surveyed at DICs and community hotspots about experiences of stigma or discrimination at public health facilities. All data collection was administered by trained healthcare service users and community members using CommCare [25]. The KP‐focused surveys were administered by KP community members.

Additionally, seven individual interviews were conducted with DIC managers from September to December 2024, using a semi‐structured interview guide. All respondents were managers of DIC “centres of excellence,” which are community‐based, led by and serving one or more KP groups, and provide clinical services such as antiretroviral (ARV) refills and viral load testing. The interview guides drew on resiliency theory [26] to identify how DICs adapted to challenges at three time periods: during legislative debate, immediately after enactment and 1 year after signing. To improve the quality of dialogue in a criminalized context, interviews were conducted by members of the CLM programme known to the respondents [27]. Interviews were conducted in English, virtually or in‐person, recorded and transcribed using automated transcription with human review [28].

The CLM programme's protocol was internally reviewed by the US Centers for Disease Control and Prevention and received an exemption from further review. The research study was approved by the Georgetown University institutional review board. Informed consent was verbally obtained from all participants.

### 2.2 Quantitative data analysis

Survey data were analysed using a difference‐in‐differences (DID) approach to evaluate changes in indicators before and after 29 May 2023, the date the AHA was signed into law, with MSM status as the exposure group for the model. Five types of surveys were analysed: clients in public health facilities, public health facility managers, DIC clients, DIC managers and KPs in community hotspots. Only public health facilities with survey responses both before and after the AHA were included in the analysis.

For each survey indicator from client‐level surveys, a logistic regression model was fit with the following equation:

$$\begin{array}{c} \log\left(\frac{\mathrm{Pr}(Y=1)}{\mathrm{Pr}(Y=0)}\right)=\beta_{0}+\beta_{AHA}\times X1+\beta_{MSM}\\ \times X2+\beta_{\left(AHA)*(MSM\right)}\times X1\times X2 \end{array}$$

Where:

- Y = CLM survey indicator
- *Β_0_* = Fixed intercept
- *β_AHA_* = Coefficient for whether the observation is before or after the AHA was signed
- *X1* = A binary variable representing whether the observation is before or after the AHA was signed (e.g. 0 = before, 1 = after)
- *β_MSM_* = Coefficient for status as MSM
- *X2* = A binary variable representing status as MSM (e.g. 0 = not MSM, 1 = MSM)
- β_(AHA)*(MSM)_ = Interaction term between pre/post AHA and MSM status

Model selection was performed using the Akaike information criterion, which was used to compare candidate models, balancing model fit and complexity. The parallel trends assumption between MSM and non‐MSM respondents was statistically tested using pre‐policy data. For survey indicators where the parallel trend assumption was violated, an interrupted time series model was additionally fit with a continuous time variable, yielding similar results to the DID models. Due to limited survey responses in the pre‐AHA period, DID was chosen as the primary modelling approach for this analysis. Models that included the interaction term demonstrated significantly improved fit compared to those with only main effects. All analyses were conducted in R, with significance assessed at a 5% level (*p*<0.05) [29].

For survey questions addressed to clients (in health clinics and DICs), the adjusted model included a binary indicator for status as MSM. In DIC surveys, respondents were directly asked to report on their KP status. Clients in public health facilities were not asked to identify as KPs. However, a proxy indicator for MSM status was calculated, with respondents categorized as MSM if they reported being male and reported being familiar with lubricants. Awareness of lubricants is hypothesized to be differentially higher among MSM, reflecting patterns of use, preferences and norms among MSM and non‐MSM in Uganda [30, 31]. For survey responses from public health facility and DIC managers, models were analysed without the coefficient for MSM status and the interaction term between MSM status and the AHA period, as MSM status was not applicable to these respondents.

Additionally, community‐based surveys that focused on stigma experienced by KPs in public health facilities were analysed. These surveys were developed in response to the AHA and, as such, only contain responses from the post period. Estimates for the proportion of affirmative responses of each indicator were calculated for both MSM respondents and other KPs, and chi‐squared tests were used to assess differences between the two.

### 2.3 Qualitative data analysis

Interviews were analysed using a theory‐driven deductive thematic approach, using a codebook developed from organizational resiliency theory with additional inductive first round coding [26, 32]. Coding was conducted in Taguette. After the first round coding, researchers grouped codes under broader components, including adaptation and coping [26]. An additional non‐resiliency‐related category describing immediate impacts of the AHA was added to understand what DICs were demonstrating resilience to. All interviews were independently coded by two researchers, with memos summarizing the broader second round codes developed and compared between coders. Researcher triangulation and broader team consensus building were used to improve analytic trustworthiness [33].

## 3 RESULTS

### 3.1 Quantitative results

Survey data were collected from 320 public health facilities and 50 DICs from 2022 to 2024, in addition to community‐based surveys on stigma and discrimination (Table 1). Respondents in facilities were mostly female (58.5%), HIV positive (80.1%) and averaged 36.9 years of age. An estimated 3.2% of respondents in facilities were categorized as being MSM, according to the proxy indicator. DIC clients were more frequently MSM (12.7%) and transgender (9.0%) as compared to health facility clients.

**Table 1:** Quantitative sample characteristics

	Facility clients	Facility managers	DIC clients	DIC managers	Stigma surveys
Survey responses	57,917	2846	1407	165	1697
Facilities/DICs surveyed	320	320	50	50	ND
Period surveyed	11/2022−08/2024	01/2022−08/2024	09/2022−08/2024	09/2022−08/2024	07/2023−09/2024
Region					
Central	16,502 (28.5%)	795 (27.9%)	638 (45.3%)^**^	83 (50.3%)^**^	ND
Eastern	18,803 (32.5%)	975 (34.3%)	175 (12.4%)	27 (16.4%)	ND
Northern	8721 (15.1%)	366 (12.9%)	160 (11.4%)	17 (10.3%)	ND
Western	13,891 (24.0%)	710 (24.9%)	122 (8.7%)	14 (8.5%)	ND
Age (mean years)	36.9	ND	ND	ND	ND
Gender					
Female	33,878 (58.5%)	ND	ND	ND	ND
Male	24,035 (41.5%)	ND	ND	ND	ND
Transgender	4 (< 0.01%)	ND	127 (9.0%)	ND	166 (9.8%)
HIV positive	46,408 (80.1%)	ND	ND	ND	ND
Key population					
MSM	1849 (3.2%)^*^	ND	178 (12.7%)	ND	202 (11.9%)
Sex worker	ND	ND	722 (51.3%)	ND	860 (50.7%)
PWUD	ND	ND	208 (14.8%)	ND	198 (11.7%)
Trans	ND	ND	127 (9.0%)	ND	166 (9.8%)
Other KP	ND	ND	159 (12.2%)	ND	271 (16.0%)

Abbreviation: ND, data were not collected.

* Proxy indicator.

** Totals do not sum to 100%, since some free‐text DIC names could not be matched to a region.

#### 3.1.1 Public health facilities

In public health facilities, several measures of healthcare access and quality improved in the period after the AHA (Table 2). These include client reports of staff friendliness (OR: 1.46, 95% CI: 1.36−1.56), having received information about pre‐exposure prophylaxis (PrEP) from a healthcare provider (OR: 1.18, 95% CI: 1.13−1.23), awareness of support groups for people living with HIV (OR: 1.13, 95% CI: 1.08−1.19), attendance of support groups (OR: 1.13, 95% CI: 1.05−1.21) and multi‐month dispensing (MMD) of ARVs (OR: 1.17, 95% CI: 1.09−1.26).

**Table 2:** Bivariate and multivariate regression coefficients for client and facility manager survey indicators

	Bivariate model OR (95% CI)	Multivariate model aOR (95% CI)		
	Pre‐post AHA β_AHA_	Pre‐post AHA β_AHA_	MSM status β_MSM_	Interaction β_(AHA)_*_(MSM)_
Clients in facilities				
Proxy for MSM status (% yes) (*n*=58,138)	**0.63 (0.57**−**0.69)** ^**^			
Staff are friendly and professional (% yes) (*n*=46,210)	**1.46 (1.36**−**1.56)** ^**^	**1.48 (1.38**−**1.58)** ^**^	0.95 (0.71−1.29)	**0.67 (0.47**−**0.93)** ^*^
Provided information on PrEP (% yes) (*n*=57,739)	**1.18 (1.13**−**1.23)** ^**^	**1.25 (1.20**−**1.30)** ^**^	**15.79 (12.47**−**20.28)** ^**^	**0.48 (0.36**−**0.64)** ^**^
Awareness of support groups (% yes) (*n*=46,210)	**1.13 (1.08**−**1.19)** ^**^	**1.12 (1.07**−**1.17)** ^**^	**1.72 (1.40**−**2.11)** ^**^	**2.16 (1.69**−**2.77)** ^**^
Have attended support groups (% yes) (*n*=18,415)	**1.13 (1.05**−**1.21)** ^*^	**1.18 (1.09**−**1.27)** ^**^	**2.19 (1.61**−**2.99)** ^**^	**0.45 (0.30**−**0.60)** ^**^
Length of ARV prescription (% 3 or more months) (*n*=45,618)	**1.17 (1.09**−**1.26)** ^**^	**1.15 (1.07**−**1.23)** ^**^	**0.29 (0.16**−**0.49)** ^**^	**2.49 (1.42**−**4.73)** ^**^
Facility managers (*n*=2846)				
Populations targeted by HIV testing services (% MSM) (*n*=2846)	**0.69 (0.59**−**0.81)** ^**^	–	–	–
Population‐specific services offered (% MSM) (*n*=2846)	**0.62 (0.52**−**0.74)** ^**^	–	–	–
Facility has a DIC (% yes) (*n*=2788)	0.89 (0.73−1.09)	–	–	–
Clients in DICs				
MSM status (% yes) (*n*=1407)	**0.44 (0.31**−**0.63)** ^*^			
Referred to health facility by DIC in last 6 months (% yes) (*n*=1395)	**0.57 (0.42**−**0.76)** ^**^	**0.58 (0.42**−**0.81)** ^**^	0.90 (0.48−1.75)	0.73 (0.34−1.52)
DIC is easy to access (% yes) (*n*=1392)	**0.20 (0.10**−**0.37)** ^**^	**0.24 (0.11**−**0.46)** ^**^	1.10 (0.27−7.48)	0.36 (0.05−1.60)
Receiving PrEP at DIC (% yes) (*n*=1331)	**0.41 (0.30**−**0.57)** ^**^	**0.48 (0.34**−**0.69)** ^**^	2.05 (0.98−4.63)	0.48 (0.20−1.11)
Tailored services provided for specific populations (% MSM) (*n*=1407)	**0.39 (0.29**−**0.51)** ^**^	–	–	–
DIC managers				
DIC provides full clinical package (% yes) (*N*=165)	**2.25 (1.07**−**4.98)** ^*^	–	–	–
Populations who receive services at DIC (% MSM) (*N*=165)	0.77 (0.32−1.69)	–	–	–

Abbreviations: aOR, adjusted odds ratio; OR, odds ratio.

* *p*<0.05.

** *p*<0.001.

Bold values indicates a statistically‐significant *p* < 0.05.

In the period after the AHA was signed, survey respondents were less likely to be MSM (OR: 0.63, 95% CI: 0.57−0.69), suggesting that MSM represented a smaller proportion of facility clients after the AHA was enacted. While MSM respondents had overall higher odds than non‐MSM respondents of being aware of support groups (aOR: 1.72, 95% CI: 1.40−2.11) and attending support groups (aOR: 2.19, 95% CI: 1.61−2.99), group attendance in the past 6 months among people who knew about support groups dropped from 63.8% in the pre‐AHA period to 46.9% in the post‐AHA period (Appendix Table S1). While overall staff friendliness increased (aOR: 1.48, 95% CI:1.39−158) after the AHA, MSM clients reported nearly no change (85.6% pre‐AHA vs. 85.4% post‐AHA). Similarly, the odds of receiving PrEP counselling increased overall (aOR: 1.25, 95% CI:1.20−1.30), but among MSM clients, the proportion of clients provided PrEP information dropped from 87.7% in the pre‐AHA period to 81.2% post‐AHA. By contrast, MSM clients were initially less likely to receive MMD compared to non‐MSM respondents (aOR: 0.29, 95% CI: 0.16−0.49) in the pre‐AHA period, but they reported a significant improvement in MMD rates in the post‐AHA period (3.5% pre‐AHA vs. 9.3% post‐AHA).

Facility managers reported significant shifts in services targeting MSM in the period after the AHA. The odds of facilities offering HIV testing services specific to MSM were 31% lower (OR: 0.69, 95% CI: 0.59−0.81), and the odds of having population‐specific services for MSM were 38% lower (OR: 0.62, 95% CI: 0.52−0.74). Managers did not report a significant shift in whether their facility operated a DIC, with 17.3% facilities having a DIC in the pre‐AHA period and 15.7% after the AHA.

#### 3.1.2 Drop‐in centres

In DICs, survey respondents were 56% less likely to self‐identify as MSM in the period after AHA went into effect. Overall, respondents were less likely in the period post‐AHA to report receiving referrals to health facilities (OR: 0.57, 95% CI: 0.42−0.76), receiving PrEP (OR: 0.41, 95% CI: 0.30−0.57) and indicate that the DIC served MSM clients (OR: 0.39, 95% CI: 0.29−0.51). Additionally, after the AHA was enacted, clients were significantly less likely to easily access a DIC (OR: 0.20, 95% CI: 0.10−0.37). None of these effects were significantly different between MSM and non‐MSM clients. Among MSM clients accessing DICs, the proportion who reported that DICs were easy to access declined 27.9 percentage points, and the proportion receiving PrEP at the DIC declined 32.0 percentage points (Appendix Table S1).

DIC managers were more likely to report providing a full package of clinical services post‐AHA (OR: 2.25, 95% CI: 1.07−4.98); however, there was no significant change in the proportion of DIC managers reporting providing services tailored to MSM clients.

Community‐based surveys of KPs revealed that 50.5% of MSM surveyed had experienced stigma in the previous 6 months at public health facilities due to their KP status, significantly higher than the 39.2% reported by other categories of KPs (Table 3). The types of stigma experienced were similar between MSM and other KPs and included rude communication (73.5% among MSM and 71.3% among other KPs) and harsh or abusive language (42.9% and 37.8%). MSM were significantly less likely than other KPs to easily find alternative service providers after experiencing discrimination. Respondents who did not return to the health facility where they experienced discrimination most commonly identified DICs as an alternative health service option.

**Table 3:** Metrics of stigma and discrimination among KPs after AHA signing

	MSM Percentage (95% CI) (number of yes responses)	Other KP	*p*‐value^*^
Faced stigma in last 6 months at a public health facility due to KP status (% yes) (*n*=1557)	**50.5% (43.5**−**57.6%) (*n* = 98**)	**39.2% (36.6**−**41.8%) (*n* = 534)**	**0.003**
Type of stigma experienced (*n*=632)			
Rude communication	73.5% (64.7−82.2%) (72)	71.3% (67.5−75.2%) (381)	0.759
Harsh or abusive language	42.9% (33.1−52.7%) (42)	37.8% (33.7−41.9%) (202)	0.408
KP identity flagged to others	21.4% (13.3−29.6%) (21)	20.2% (16.8−23.6%) (108)	0.892
Conversion therapy language from healthcare worker	12.2% (5.8−18.7%) (12)	20.6% (17.2−24.0%) (110)	0.074
Denied service due to identity	13.3% (6.5−20.0%) (13)	13.9% (10.9−16.8%) (74)	1.000
Unwanted physical contact	14.3% (7.4−21.2%) (14)	12.9% (10.1−15.8%) (69)	0.838
Aware of a system to report discrimination (*n*=998)	28.8% (20.4−37.3%) (32)	36.0% (32.8−39.1%) (319)	0.168
Facility staff have shared KP status with someone outside facility (% yes) (*n*=1559)	8.2% (4.4−12.1%) (16)	7.3% (5.9−8.7%) (100)	0.755
After experiencing discrimination, returned to health facility (% yes) (*n*=632)	31.6% (22.4−40.8%) (31)	42.5% (38.3−46.7) (227)	0.057
Other options available for services, among people who did not return to health facility (*n*=374)			
Drop‐in centre	53.7% (41.8−65.7%) (36)	55.7% (50.1−61.3%) (171)	0.874
Private hospital	38.8% (27.1−50.5%) (26)	31.3% (26.1−36.5%) (143)	0.295
Public health facility in another district	3.0% (0−7.1%) (2)	7.2% (4.3−10.1%) (22)	0.322
No other option	4.5% (0−9.4%) (3)	5.9% (3.2−8.5%) (18)	0.878
Consider it easy to find other options for treatment as a KP (*n*=1697)	**27.7% (21.5**−**33.9%) (56)**	**43.9% (41.4**−**46.5%) (657)**	**<0.001**

Bold values indicates a statistically‐significant *p* < 0.05.

* The *p*‐values reported are from chi‐squared tests used to assess the statistical significance of differences in stigma‐related indicators between MSM and other KPs.

### 3.2 Qualitative results

#### 3.2.1 Disruptive effects of the AHA on DICs

According to interviewed DIC managers, the AHA led to increases in harassment and vigilantism, difficulties maintaining affordable and safe spaces, challenges with human resources and staff wellbeing, clients dropping out of care, and disruption to supply chain and referral networks. These experiences extended beyond the LGBTQ+‐led DICs, impacting other KP‐led sites, which did not expect to be targeted by community violence and thus felt less prepared.

“*At the back of our mind, we were like, ah, [the AHA is] it's not going to affect sex workers. It's for the other KPs…we were a bit reluctant to mobilize, to strengthen, to sensitize our communities, to be resilient to, to be prepared, to just to know that in case this happens or just in case this is happening this is what we need to do. So we didn't do that, I must say. But the assaults still happened [to us]*.”

DIC managers reported harassment, including stalking of staff, verbal threats, radio and social media efforts that publicly identified DICs and encouraged action against them, and physical violence. Community vigilantism was frequently described, with individuals pretending to be clients at DICs with the aim of uncovering criminalized behaviour. Managers more commonly reported arrests among community‐based peer‐volunteers and educators than DIC‐based staff.

*“A lot of unknown people [were] coming into our DIC to ask about our work. And you could clearly see that these were not even service beneficiaries, but they had very weird questions that they were posing to our staff. […] The [KPs used to] feel like [the DIC] was a safe space for them. But when these anonymous people started coming in asking questions […] what they usually refer to as their safe space was now turning into a place where they had to be more vigilant and to be very careful, [which] eventually started reducing the numbers that were coming in to receive services at that [DIC].”*

Issues with landlords and maintaining a physical space for the DIC were commonly reported. These included reports of landlords selling property, denying the sale to the DIC and raising rent substantively, which were perceived as intentional efforts to displace the DICs. Some DIC managers described their spaces being vandalized by community members, while others experienced government raids and inspections where patient records were confiscated. Safety threats and harassment extended beyond the confines of the DIC site, with one staff member forced out of their housing because of their association with the DIC, and several DIC staff relocated because of threats. Respondents reported that family members of DIC staff were also regularly targeted.

*“Running a [DIC], especially in this legal environment right now, I think at the heart of it is the heart you have for the community, because it takes a lot. It takes a lot on your mental health. It takes a lot to live in constant fear every single day. Because for us, every single day, we walk into that [DIC], we will either live or die sitting here.”*

Some DIC staff quit in response to harassment and threats. Since most staff were members of KP groups, participants described the dual challenges of living as a member of a criminalized population while also providing services in a hostile climate. DIC managers noted that their staff providing peer‐to‐peer services were particularly vulnerable, since their work involved more visible, community‐based outreach. In some cases, peer workers were described as being too afraid to do their work, or the DIC leadership considered it too dangerous to continue. Some respondents reported significant mental health challenges, with DICs often lacking resources to support their psychosocial wellbeing.

*“Human resources also declined. We saw a lot of people [with] suicidal thoughts. For example, half of our staff, or human resources went…they went silent. You reach out to them, they are not responding.”*

DIC managers noted that many KPs stopped attending DICs for services, which was observed both while the AHA was being discussed and to a greater extent after the signing and was true of LGBTQ+‐led DICs and non‐LGBTQ+‐led DICs. DIC managers reported challenges reengaging people who had fallen out of care, given an overall climate of fear, with KP clients not responding to calls or relocating to relatives’ homes outside of Kampala, where they were less known to the community. DIC managers described an increase in HIV diagnoses, as well as the deaths of several people living with HIV, who had disengaged from care out of fear of being seen.

*“[Y]ou would hear people calling you, come this way, so‐and‐so is dead. Why? You know, she fell sick. She was scared to come [get services] and instead she went back to the village. So, people chose to actually get sick.”*

*“It was like a match box they put on the dry forest, the fire started, children were thrown out of homes, children were being attacked in the communities. People going to facilities said, ah, let me die. Because if I go there, they will start seeing me and they might actually report me because most of us […] in the communities we did not understand again the effects of the impact of the ruling, of some of the specific word clauses.”*

DICs described confusion from clients and staff around how the AHA impacted the legality of delivering and receiving services. The 2023 MOH circular, which mandated non‐discriminatory health services for all, was interpreted by many respondents to mean that healthcare providers would not be at risk of arrest when providing services to LGBTQ+ individuals. However, since most DICs were not registered as clinics prior to the AHA, DIC managers described feeling unprotected by the circular.

The unique role of DICs as quasi‐clinical spaces made them vulnerable after the AHA was signed. DIC managers reported that some DICs provided clinical services, while others relied on informal referral networks with public health facilities. After the AHA, managers described some of these relationships as breaking down when clinicians began refusing to work with DICs due to perceived legal risk. Managers also reported that DICs reliant on informal arrangements with public health facilities lost access to commodities, particularly lubricants, which became widely unavailable. The Ugandan supply chain was concurrently facing challenges, so respondents lacked visibility into whether procurement issues were directly related to the AHA.

*“Nobody knew what to do at the time. Even our own allies would say…we know you people. We know the work you're doing. But we are also scared because this could also implicate us. This could also affect our funding. This could also affect our reputation. So, we felt like almost everyone had abandoned us at the time.”*

#### 3.2.2 Coping with the AHA immediately post‐signing

In parallel to the acute challenges experienced after the AHA enactment, DIC managers reported several innovative strategies for continuing services. These included piloting hybrid forms of service delivery, serving as conduits for patients unable to access health facilities and building support through partnerships. Respondents felt these strategies enabled most of the DICs that closed or significantly reduced service provision immediately after AHA to reopen in some capacity several weeks or months later.

Every DIC manager interviewed reported adaptations to their operating procedures to be able to provide services more safely. Given the threat of arrest, several DICs returned to preestablished protocols from the COVID‐19 lockdowns, where virtual care was preferentially used. One DIC reported ending drop‐in services and instead scheduling appointments at irregular times of the week to reduce predictability and thus the risk of raids and community threats.

*“For example, we used to work from morning and evening. So, we decided okay, today I want to come in around 10:00 and leave at 2:00, so that actually if there's anyone that is following us, they don't know the proper time that [the DIC] is really operational.”*

DIC managers felt their sites served as important bridges with public health facilities when clients were too fearful to attend. One DIC collected blood samples for clients and delivered them to the facility on their behalf. In another case, a DIC manager reported providing a home delivery service for condoms and lubricants, despite several other DICs reportedly being unable to stock lubricants on site due to their perceived association with homosexuality.

Respondents described partnerships as playing a role in adapting to the post‐AHA context. While some district health officials began refusing to work with LGBTQ+‐led DICs, respondents reported that others supported DICs with safety assessments and by expediting the process to register DICs as clinics. Respondents reported that PEPFAR facilitated strategic planning before the AHA among KP providers, and that its implementing partners at times intervened on behalf of DICs with local government and others. Multiple DIC managers also noted they received financial support to adapt; for example, DICs received support from PEPFAR's LIFT UP fund to upgrade security systems and install cameras and biometric locks. However, respondents described emergency funds arriving after they were most needed.

According to DIC managers, local community leaders, such as district council members, were allies in mitigating conflict between DICs and the community, curbing vigilante activity and responding to landlord extortion. Staff from public health facilities and NGOs partnered with DICs, acting as a protective “shield.” One DIC reported resuming community outreach when staff from a partner clinic joined their efforts, leveraging their status to protect DIC personnel.

“[During community outreach] we had to […] be in presence of someone from your supporting health facility […] because none of us has an ID that mentions you being a staff of a health facility. We had to wait on a staff from on a staff from [Health Facility] to be part of our team during our community outreaches.”

Another DIC invited partner organizations to events, believing their presence would deter raids and arrests. To appear less “suspicious,” one LGBTQ+‐led DIC began offering services to sex workers. Respondents additionally described non‐clinical Ugandan LGBTQ+ organizations helping DICs relocate threatened staff and clients and provided essential emergency support, including legal referrals.

#### 3.2.3 Longer‐term adaptation to the AHA's effect on DICs

One year after AHA's enactment, the long‐term effects and strategies for adaptation remained unclear. DIC managers continued to describe harassment and raids, and most reported that client numbers remained below pre‐AHA levels. Nonetheless, many DIC adaptation strategies were seen as successful by respondents, improving client retention and commodity procurement.

DIC managers reported that their sites are implementing longer‐term strategies to strengthen their resiliency. One DIC that was forced out of its rental space reported leading a grassroots fundraising effort that allowed it to purchase a land parcel for its operations. Managers reported that some DICs reliant on informal referral and procurement partnerships negotiated written contractual arrangements. Respondents reported that since the accreditation process for DICs was never formalized, some DICs chose to become registered clinic sites to receive protection under the MOH circular. Some DICs chose not to become registered, preferring to serve a non‐clinical role; others feared an administrative process that would legally expose them.

*“By the time they signed the AHA, we got a communication from the local government and they're like, ‘you guys, do you know what? Now the best way to protect your DIC is by legalizing it. Let's get you the papers, and we'll register it as a clinic.’ […] By [mid‐June] we had been certified […] and they had legalized our DIC as a clinic.”*

One service delivery change reported by multiple DIC managers was the strengthening of psychosocial support services offered to their staff and clients, either by providing additional virtual offerings or introducing new counselling and support group services. Respondents described that these services were still maintained more than a year after the AHA signing.

*“If someone is traumatized by a sudden action of someone who is violating their human rights, it still comes back to their mental health and it takes a toll on their mental health. So we provide mental health awareness […] That is one of our new services that runs once every month, where community members come together to share experiences in this community, […] as people who are living in extreme marginalization and criminalisation.”*

## 4 DISCUSSION

This analysis used community‐generated data to measure the impact of the AHA on HIV‐related service delivery in public health facilities and DICs in Uganda. We found that the AHA had a chilling effect on service use and provision in both DICs and public health facilities, driven by an increase in discrimination, stigmatization, harassment and fear of arrest. These data align with a limited but emerging body of literature on the AHA's impact, which has found declines in KP visits to PEPFAR‐supported DICs, particularly in the period when the bill was originally introduced, as well as widespread forced evictions, discrimination and assault directed at LGBTQ+ people [34, 35]. While this study does not directly measure epidemiological outcomes, the observation of clients dropping out of treatment and dying from HIV‐related causes is consistent with previously described associations between criminalization and lower rates of status awareness, higher HIV prevalence, and lower viral suppression [17, 18, 36].

These data suggest that the effects of the AHA extended beyond legal consequences from government actors to include extrajudicial intimidation from the community. This finding is corroborated by an analysis in Uganda that found that “extra‐state vigilantes” were as much of a barrier to healthcare delivery as were the legal restrictions themselves [37]. In Ghana, criminalization has led to a greater normalization of non‐state violence against LGBTQ+ persons, regardless of the level of government enforcement of the legislation [38]. Importantly, one key finding was that the impacts of criminalization on healthcare were experienced by other KP groups and not solely LGBTQ+ clients.

These findings describe DICs as an important, but uniquely vulnerable, safety net for criminalized populations. After the AHA's enactment, HIV services in public health facilities became less tailored to the needs of MSM, less friendly and less accessible. After experiencing high levels of stigma and discrimination in these clinics, KPs were most likely to identify DICs as an alternative space to receive care, even while the DICs themselves struggled to continue providing accessible services. This finding is consistent with data from Kenya [39] and Tanzania [40], which find that KP‐led service delivery is preferable for LGBTQ+ individuals in criminalized and highly stigmatized settings. However, DICs were perceived to be more exposed to legal consequences, being quasi‐clinical spaces without government protection. Additionally, as informal relationships between DICs and health facilities deteriorated, DICs struggled with providing referrals and commodities to their clients.

These findings have policy implications for HIV provision in highly criminalized contexts. First, criminalization undermines the global HIV response, making the repeal of the AHA an urgent priority for human rights and public health. Second, the MOH's efforts to mandate non‐discriminatory care have failed to address stigmatization and discrimination, highlighting the need for better communication about healthcare providers’ legal duties under the AHA. Third, initiatives by PEPFAR, the United States Agency for International Development (USAID), and others to mitigate the bill's impact have been effective and should be strengthened. Given high levels of disengagement from care, this support must also include intensive follow‐up to re‐engage person/people living with HIV (PLHIV) in care. Finally, key strategies to protect DICs and KP‐focused services include registering DICs as legally recognized clinics, finalizing the accreditation process for DICs by the MOH, formalizing procurement processes, and improving referrals between DICs and public health facilities.

This analysis is to our knowledge the first to describe the impacts of criminalization on HIV services using community‐owned data. In Uganda, the CLM data collection is led by trusted members of the community, enabling a level of access to marginalized and vulnerable populations less feasible through traditional research methods.

### 4.1 Limitations

This study had several limitations. First, data were taken from the Uganda CLM programme, which gathers data for accountability‐focused advocacy, not research. As such, randomization techniques were not used when sampling sites and respondents, and distinct cohorts were surveyed in the two periods. We hypothesize that this bias could underrepresent barriers in our analysis, since the sample of clients who continued to receive care in clinics and DICs, and were, therefore, accessible to the CLM programme's data collection, are likely to have experienced fewer barriers to care than those who disengaged from services entirely. Additionally, due to the AHA context, respondents in health facilities were not asked to identify their KP status; as such, the MSM metric calculated in the analysis is a functional but imperfect proxy. The quantitative analysis also did not measure the impact of AHA on other LGBTQ+ populations, including transgender people and women who have sex with women.

The sample sizes of respondents in DICs were small, although sizable for a sample of stigmatized respondents in a criminalized context. The qualitative sample only represented experiences from managers of DIC centres of excellence. Since these are typically larger and more established than other DICs, they may have experienced more vigilantism than other DICs, may have had more partners and external support during the AHA or both. Finally, the stigma‐related surveys were only available post‐AHA and the questions were not formally validated, which may limit the comparability of our findings.

## 5 CONCLUSIONS

The AHA portends a concerning global trend towards the criminalization of sexual minorities. Several countries, including Burundi, Ghana and Kenya, have either adopted or are in the process of adopting similar legislation. Measuring the impact of criminalization on the HIV response, and documenting strategies used by healthcare systems to mitigate harm, is urgently needed. This analysis uses community data to highlight disruptions in service delivery, the chilling effect on access to care and the approaches taken to restore continuity of care. Ensuring equitable access to healthcare services in criminalized contexts is a public health and human rights priority.

## COMPETING INTERESTS

The authors have no conflicts of interest to declare.

## AUTHOR CONTRIBUTIONS

AN, BA, KM and JK designed the CLM programme methodology, designed the data collection indicators and collected the data. ARS, EL and PD designed the data analysis protocol. ARS, EL and PD analysed the data. PD conducted statistical analyses. AN, ARS, EL and PS led the writing of the paper. BA, FM, KM, EJK and AK contributed to the writing of the paper.

## FUNDING

The Uganda CLM programme is supported by the United States Centers for Disease Control and Prevention (Washington, DC). Support for the manuscript development was provided by the International AIDS Society (Geneva).

## Supporting information




**Table S1**: Mean and confidence interval estimates for client and facility manager survey indicators, by pre‐ and post‐AHA

## Data Availability

The Uganda CLM programme publishes facility‐level survey data online, which are available for download (http://clmdev.amfar.org/UG). Data from DICs are not available on the website due to safety concerns for clients and staff. The DIC data are available on request from the corresponding author. The interview audio files and transcripts used in this analysis cannot be shared publicly per the CLM programme protocol and safety concerns. However, an analysis summary table may be available upon reasonable request to the corresponding author and with the permission of the CLM programme.
