# Supplementary material for: Impact of the Anti‐Homosexuality Act on HIV service delivery in Uganda: Evidence from community‐led monitoring
Source: J Int AIDS Soc. 2025 Sep 12;28(9):e70030. doi: 10.1002/jia2.70030 (PMC12432269; doi:10.1002/jia2.70030)
Supplement: Supplementary file 1 — Table S1: Mean and confidence interval estimates for client and facility manager survey indicators, by pre‐ and post‐AHA [file JIA2-28-e70030-s001.docx]

**Supplemental Table 1. Mean and confidence interval estimates for client and facility manager survey indicators, by pre- and post-AHA**

| **Clients in Facilities** | **Pre-AHA (MSM)** | **Post-AHA (MSM)** | **Pre-AHA (Non-MSM)** | **Post-AHA (Non-MSM)** |
| --- | --- | --- | --- | --- |
| Proxy for MSM status (% yes) (n=58,138) | 4.4% (4.0-4.7%) | 2.8% (2.6-2.9%) | – | – |
| Staff are friendly and professional (% yes) (n=46,210) | 85.6% (82.1-89.2%) | 85.4% (83.3-87.6%) | 86.3% (85.6-86.9%) | 90.3% (90.0-90.6%) |
| Provided information on PrEP (% yes) (n=57,739) | 87.7% (85.1-90.3%) | 81.2% (79.0-83.4%) | 31.2% (30.4-31.9%) | 36.1% (35.7-36.6%) |
| Awareness of support groups (% yes) (n=46,210) | 50.5% (45.5-55.6%) | 71.2% (68.4-74.0%) | 37.3% (36.3-38.3%) | 40.0% (39.5-40.5%) |
| Have attended support groups (% yes) (n=18,415) | 63.8% (56.9-70.7%) | 46.9% (43.2-50.6%) | 44.6% (43.0-46.2%) | 48.7% (47.8-49.5%) |
| Length of ARV prescription (% 3 or more months) (n=45,618) | 3.5% (1.6-5.3%) | 9.3% (7.5-11.1%) | 10.9% (10.3-11.5%) | 12.3% (11.9-12.6%) |
| **Facility managers** | **Pre-AHA** | **Post-AHA** |  |  |
| Populations targeted by HIV testing services (% MSM) (n=2,846) | 37.9% (35.2-40.6%) | 29.8% (27.6-32.0%) | – | – |
| Population-specific services offered (% MSM) (n=2,846) | 26.9% (24.4-29.4%) | 18.6% (16.7-20.5%) | – | – |
| Facility has a DIC (% yes) (n=2,788) | 17.3% (15.2-19.4%) | 15.7% (14.0-17.5%) | – | – |
| **Clients in DIC** | **Pre-AHA (MSM)** | **Post-AHA (MSM)** | **Pre-AHA (Non-MSM)** | **Post-AHA (Non-MSM)** |
| MSM status (% yes) (n=1,407) | 21.6% (16.4-26.8%) | 10.8% (9.0-12.6%) | – | – |
| Referred to health facility by DIC in last 6 months (% yes) (n=1,395) | 65.4% (52.5-78.3%) | 44.4% (35.8-53.1%) | 67.7% (61.1-74.4%) | 55.1% (52.0-58.1%) |
| DIC is easy to access (% yes) (n=1,392) | 96.2% (90.9-100.0%) | 68.3% (60.1-76.4%) | 95.8% (92.9-98.6%) | 84.3% (82.1-86.5%) |
| Receiving PrEP at DIC (% yes) (n=1,331) | 79.6% (68.5-90.7%) | 47.6% (38.8-56.3%) | 65.6% (58.8-72.4%) | 47.9% (44.9-50.9%) |
| Tailored services provided for specific populations (% MSM) (n=1,407) | 96.2% (90.9-100.0%) | 93.7% (89.4-97.9%) | 54.5% (47.4-61.6%) | 33.7% (30.8-36.5%) |
| **DIC managers** | **Pre-AHA** | **Post-AHA** |  |  |
| DIC provides full clinical package (% yes) (N=165) | 30.8% (16.1-45.4%) | 50.0% (41.2-58.8%) | – | – |
| Populations that receive services at DIC (% MSM) (N=165) | 74.4% (60.5-88.2%) | 69.0% (60.9-77.2%) | – | – |
